# Convulsive liability of bupropion hydrochloride metabolites in Swiss albino mice

**DOI:** 10.1186/1744-859X-7-19

**Published:** 2008-10-15

**Authors:** Peter H Silverstone, Robert Williams, Louis McMahon, Rosanna Fleming, Siobhan Fogarty

**Affiliations:** 1 Biovail Corporation, Mississauga, Ontario, Canada; 2 Biovail Technologies Ltd, Dublin, Ireland; 3 Statistical Group, Biovail Technologies Ltd, Bridgewater, NJ, USA

## Abstract

**Background:**

It is known that following chronic dosing with bupropion HCl active metabolites are present in plasma at levels that are several times higher than that of the parent drug, but the possible convulsive effects of the major metabolites are not known.

**Methods:**

We investigated the convulsive liability and dose-response of the three major bupropion metabolites following intraperitoneal administration of single doses in female Swiss albino mice, namely erythrohydrobupropion HCl, threohydrobupropion HCl, and hydroxybupropion HCl. We compared these to bupropion HCl. The actual doses of the metabolites administered to mice (n = 120; 10 per dose group) were equimolar equivalents of bupropion HCl 25, 50 and 75 mg/kg. Post treatment, all animals were observed continuously for 2 h during which the number, time of onset, duration and intensity of convulsions were recorded. The primary outcome variable was the percentage of mice in each group who had a convulsion at each dose. Other outcome measures were the time to onset of convulsions, mean convulsions per mouse, and the duration and intensity of convulsions.

**Results:**

All metabolites were associated with a greater percentage of seizures compared to bupropion, but the percentage of convulsions differed between metabolites. Hydroxybupropion HCl treatment induced the largest percentage of convulsing mice (100% at both 50 and 75 mg/kg) followed by threohydrobupropion HCl (50% and 100%), and then erythrohydrobupropion HCl (10% and 90%), compared to bupropion HCl (0% and 10%). Probit analysis also revealed the dose-response curves were significantly different (p < 0.0001) with CD_50 _values of 35, 50, 61 and 82 mg/kg, respectively for the four different treatments. Cox proportional hazards model results showed that bupropion HCl, erythrohydrobupropion HCl, and threohydrobupropion HCl were significantly less likely to induce convulsions within the 2-h post treatment observation period compared to hydroxybupropion HCl. The mean convulsions per mouse also showed the same dose-dependent and metabolite-dependent trends.

**Conclusion:**

The demonstration of the dose-dependent and metabolite-dependent convulsive effects of bupropion metabolites is a novelty.

## Background

The pharmacology underlying the convulsive liability of bupropion hydrochloride (HCl) is not known. Furthermore, it is not known whether the seizure risk of bupropion is due principally to the parent drug or to one of its three active metabolites or to a combination of more than one of the four [1]. However, it is well established that: (1) bupropion is extensively metabolized in humans to 3 active metabolites, hydroxybupropion, threohydrobupropion, and erythrohydrobupropion; (2) following administration of bupropion HCl in humans, the areas under the plasma concentration-time curves (AUCs) at steady-state for hydroxybupropion, threohydrobupropion, and erythrohydrobupropion are 17 times, 7 times and 1.5 times higher, respectively, than the AUCs of the parent drug for the immediate release (IR) formulation, and 13 times, 7 times and 1.4 times higher, respectively, than the AUCs of the parent drug for the once-daily extended-release (Wellbutrin XL, Biovail Technologies Ltd. 3701 Concorde Parkway Chantilly, Virginia, USA) formulation; and (3) after chronic dosing, the elimination half-life, t_0.5_, of threohydrobupropion and erythrohydrobupropion (33 ± 10 and 37 ± 13 h, respectively) are longer than that of the parent drug (21 ± 9 h), while that of hydroxybupropion is similar to that of the parent drug [2-4]. Since the seizure risk of bupropion is dose-dependent [2-11] and, hence, concentration-dependent, the latter pharmacokinetic parameters of the metabolites and parent drug of bupropion HCl may be related to its seizure risk.

Although there are a few reports on the relative contribution of the metabolites to the antidepressant effects of bupropion [2-4,12-15] and the therapeutic effect of bupropion is presumed to be due in part to the antidepressant activity of the three active metabolites [14], by contrast there are no studies of the relative contribution of each of these active metabolites to the epileptogenic effects of bupropion [1]. Therefore, the objective of this study was to investigate the convulsive liability and dose-response of single doses of bupropion metabolites and bupropion HCl administered intraperitoneally in Swiss albino mice.

## Materials and methods

The study protocol and any amendment(s) or procedures involving the care and use of animals were reviewed and approved by Charles River Laboratories Preclinical Services Inc.'s (CRM) Institutional Animal Care and Use Committee (Charles River Canada Inc., St Constant, Quebec, Canada). During the study, the animals were maintained in a facility fully accredited by the Standards Council of Canada (SCC) and the care and use of the animals was conducted in accordance with the guidelines of the Canadian Council on Animal Care (CCAC).

### Animals

A total of 120 female Swiss Crl: CD1 (ICR) albino mice (*Mus Musculus*; Charles River) of approximately 7 weeks of age, and weighing 22.9 to 31.7 g were housed individually in stainless steel wire mesh-bottomed cages equipped with an automatic watering valve in an environmentally controlled animal room (temperature 22 ± 3°C; relative humidity 50 ± 20%) with a 12-h light/dark cycle. Each animal was uniquely identified using an indelible marker and each cage was clearly labeled with a color-coded cage card indicating group, animal number and sex. All animals were acclimated to their cages and to the light/dark cycle for a minimum period of 8 days prior to the initiation of treatment. In addition, all animals had free access to a standard certified pelleted commercial laboratory diet (PMI Certified Rodent Diet 5002; PMI Nutrition International Inc., St Louis, MO, USA) and tap water except during designated procedures. Prior to the initiation of treatment, animals were randomly assigned to 12 single-dose treatment groups of 10 mice per group, using a computer-based randomization procedure that ensures stratification by body weights as follows: group 1: bupropion HCl 25 mg/kg by intraperitoneal (IP) injection; group 2: bupropion HCl 50 mg/kg IP; group 3: bupropion HCl 75 mg/kg IP; group 4: erythrohydrobupropion HCl 25 mg/kg IP; group 5: erythrohydrobupropion HCl 50 mg/kg IP; group 6: erythrohydrobupropion HCl 75 mg/kg IP; group 7: threohydrobupropion HCl 25 mg/kg IP; group 8: threohydrobupropion HCl 50 mg/kg IP; group 9: threohydrobupropion HCl 75 mg/kg IP; group 10: hydroxybupropion HCl 25 mg/kg IP; group 11: hydroxybupropion HCl 50 mg/kg IP; group 12: hydroxybupropion HCl 75 mg/kg IP. Animals in poor health or at the extremes of the prespecified body weight range (18–30 g) or those considered unsuitable for use in the study were not assigned to treatment groups and unassigned animals were released from the study.

### Drugs

Bupropion HCl was obtained from Biovail Corporation (Steinbach, Manitoba, Canada), in white powder form, with 100.3% purity, lot number RM0400, and was stored at room temperature and protected from light.

Erythrohydrobupropion HCl (lot number 200695), Threohydrobupropion HCl (lot number 200694), and Hydroxybupropion HCl (lot number 200696) were each obtained from Biovail Corporation in white powder form, with > 97% purity, and were stored frozen and protected from light.

Vehicle was 0.9% sodium chloride (NaCl) for injection United States Pharmacoepia (USP) and was obtained from Baxter Healthcare Corporation (Deerfield, IL, USA) in clear liquid form, lot number W6J12C2.

The dose formulations of bupropion HCl and the metabolites were prepared on each day of dosing. The appropriate amount of bupropion HCl or metabolite (erythrohydrobupropion HCl or threohydrobupropion HCl or hydroxybupropion HCl) was weighed into a suitable container to achieve the required dose concentration of each compound. The bupropion metabolites were adjusted to be equimolar to bupropion HCl and based on their molecular weights, 100.7 mg, 100.7 mg and 105.8 mg of erythrohydrobupropion HCl, threohydrobupropion HCl and hydroxybupropion HCl, respectively, were equivalent to 100 mg of bupropion HCl. An appropriate volume of 0.9% NaCl was added and the formulation was vortexed until the material was completely dissolved. Lower dose concentrations (solutions) of each compound were then prepared by dilution of the highest dose concentration with 0.9% NaCl. The dose formulations were kept at room temperature and protected from light. On each day of treatment, bupropion HCl or metabolite was administered by IP injection in a dose volume of 10 ml/kg and dose concentration of either 2.5, 5 or 7.5 mg/ml for the 25, 50, and 75 mg/kg doses, respectively. The actual dose administered was based on the most recent body weight of each animal.

### Study procedure

All animals were examined twice daily for signs of ill health following arrival and prior to the initiation of treatment, except on the day of arrival when they were examined only once. After the acclimation period and randomization, on the day prior to the initiation of treatment, all animals were weighed and the individual body weights were used for dose volume calculation. Single-dose IP treatment was then initiated and lasted for 6 consecutive days with equal numbers of animals from each group dosed on each day. Following treatment, all animals were observed continuously for the occurrence of convulsions for a period of 2 h along with a 5-min assessment at 24 h post dose. Animals were placed in clear Perspex observation boxes during the observation periods. During the observation periods, details of the number, time of onset, duration and the intensity of the convulsions were recorded. The duration of each convulsion was graded as short (1 to 10 s), medium (11 to 30 s), or long (≥ 31 s). The intensity of each convulsion was graded using the Charles River Laboratories grading system of either mild, moderate, or severe defined as follows: mild = head and tail slightly extended and little jerking; moderate = head and tail fully extended and some jerking; severe = head and tail fully extended and strong jerking.

In addition, the presence or absence of ataxic gait, paralysis, and catatonic episodes (without a grading of the intensity or number) were recorded over each 15 min observation period. Any animal that had a single episode of severe seizure lasting longer than 1 min or any animal displaying greater than 40 separate episodes of severe convulsions over a 1-h period was killed for humane reasons. At the end of the study, all animals were killed using humane methods.

### Assessment of convulsant activity

The primary outcome variable was the percentage of convulsing mice following treatment. This was the number of animals with convulsions divided by the total number of animals in each group multiplied by 100. The secondary outcome variables were the time to onset of convulsions, mean ± SD convulsions per mouse in each group, the duration of convulsions, and the intensity of convulsions.

### Statistical analysis

The study data were summarized and tabulated by treatment group for the primary outcome variable, the percentage of convulsing mice, and the four secondary outcome variables including, the time to onset of convulsions, mean ± SD convulsions per mouse in each group, duration of convulsions, and the intensity of convulsions. The CD_50 _values for each treatment group were calculated using the Probit procedure in SAS (SAS Institute, Cary, NC, USA). The 95% confidence limits for the CD_50 _values were calculated according to the method of Litchfield and Wilcoxon [16]. However, only the CD_50 _for the erythrohydrobupropion HCl group had 95% confidence intervals because the other groups lacked data points between 0% and 100%. Also, the dose-response curves for the treatments were compared using Probit analysis. Time to onset of first convulsion was analyzed using the Cox proportional hazards model with dose and treatment as predictors. The mice that did not have convulsions during treatment and by the end of the 2-h post treatment observation period were treated as being censored at 120 min. Because the number of events (convulsing mice/convulsions) following treatment with bupropion HCl was small and all ten mice (100%) in the two dose groups in which convulsions were observed in the study had convulsions following hydroxybupropion HCl treatment, the hydroxybupropion HCl metabolite treatment was used as the control treatment to which the individual times to onset of first convulsion obtained for the other treatments were compared. The number of convulsions per mouse was analyzed using an analysis of variance (ANOVA) model. The model contained factors for treatment and dose. A p value of less than 0.05 was considered statistically significant.

## Results

Of the 120 mice dosed in the study, 46 had convulsions. The IP administration of single doses of bupropion HCl and erythrohydrobupropion HCl 25, 50 and 75 mg/kg were not associated with any deaths in mice. Similarly, no deaths occurred following the administration of 25 and 50 mg/kg doses of threohydrobupropion HCl and hydroxybupropion HCl. However, three mice were killed for humane reasons within 15 min of dosing with threohydrobupropion HCl 75 mg/kg, and four mice and one mouse within 30 min and 45 min, respectively, of dosing with hydroxybupropion HCl 75 mg/kg. In addition, clinical signs, including ataxic gait, catatonia, changes in activity, and changes in respiration were observed with all treatment groups. Both mortality and the observed clinical signs in mice were dose-dependent and metabolite-dependent with the least number of deaths and least intense clinical signs occurring following bupropion HCl treatment and the highest number of deaths and most intense clinical signs occurring following hydroxybupropion HCl treatment (bupropion HCl < erythrohydrobupropion HCl < threohydrobupropion HCl < hydroxybupropion HCl treatment).

### Percentage of convulsing mice

**Figure 1:**
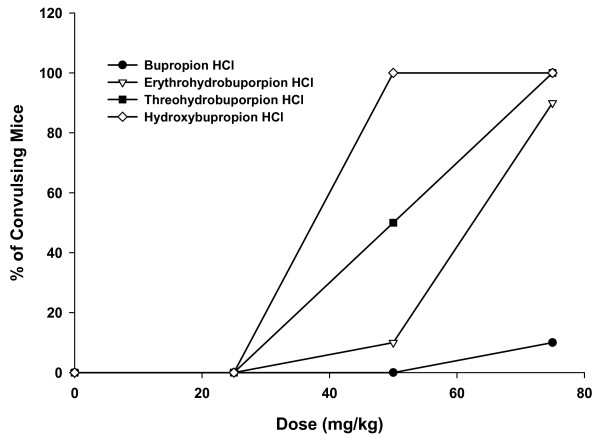
**Dose-response curves of the percentage of convulsing mice vs single doses of intraperitoneal bupropion HCl, erythrohydrobupropion HCl, threohydrobupropion HCl and hydroxybupropion HCl 25, 50 and 75 mg/kg in Swiss albino mice**. Probit analysis revealed the dose-response curves were statistically significantly different (p < 0.0001) and the CD_50 _values were 82, 61, 50 and 35 mg/kg for the bupropion HCl, erythrohydrobupropion HCl, threohydrobupropion HCl and hydroxybupropion HCl treatments, respectively. The actual doses of the three metabolites administered to mice were equimolar equivalents of the doses of bupropion HCl 25, 50 and 75 mg/kg. HCl = hydrochloride.

The IP administration of treatments induced convulsions in mice in a dose-dependent manner with each treatment (Table 1 and Figure 1). The 25 mg/kg dose for all treatments as well as bupropion HCl 50 mg/kg dose did not induce any convulsions in mice. Furthermore, the convulsions induced by the treatments were metabolite-dependent with the hydroxybupropion HCl treatment inducing the largest percentage of convulsing mice (50 mg/kg = 100%; 75 mg/kg = 100%) followed by threohydrobupropion HCl (50 mg/kg = 50%; 75 mg/kg = 100%), then erythrohydrobupropion HCl (50 mg/kg = 10%; 75 mg/kg = 90%), and bupropion HCl (50 mg/kg = 0%; 75 mg/kg = 10%).

**Table 1:** Percentage of convulsing mice following the intraperitoneal administration of bupropion HCl and bupropion metabolites in mice

Dose* (mg/kg)	**Bupropion HCl**	**Erythrohydrobupropion HCl**	**Threohydrobupropion HCl**	**Hydroxybupropion HCl**
25	0%	0%	0%	0%
50	0%	10%	50%	100%
75	10%	90%	100%	100%

*10 mice per dose per treatment.

The CD_50 _values were 35, 50, 61 and 82 mg/kg for the hydroxybupropion HCl, threohydrobupropion HCl, erythrohydrobupropion HCl and bupropion HCl treatments, respectively. In addition, Probit analysis revealed that the dose-response curves for the four treatments (Figure 1) were statistically significantly different (p < 0.0001) from each other.

### Time to onset of convulsions

The shortest times to onset of first convulsion observed in the study following treatment were 2 min in one mouse and 3 min in three mice, all in the hydroxybupropion HCl 75 mg/kg dose group. Of the 46 mice that had convulsions in the study, the times to onset of first convulsion post dosing were less than 10 min in 40 mice. The observed longest time to onset of first convulsion of 62 min was recorded in one mouse in the erythrohydrobupropion HCl 75 mg/kg dose group.

Table 2 shows the results of three Cox proportional hazards models comparing hydroxybupropion HCl to each of the other three treatments. The hazard ratios of 0.006 (p < 0.0001), 0.20 (p = 0.0002) and 0.47 (p = 0.0310) observed for the bupropion HCl, erythrohydrobupropion HCl and threohydrobupropion HCl treatments, respectively, indicate that compared to hydroxybupropion HCl, the three treatments were 99.4%, 80% and 53%, respectively, significantly less likely to induce convulsions within the 120 min post dose observation period. In addition, the hazard ratio obtained for dose was a consistent and significant value of 1.1 (p < 0.0001 for each) for all three treatments, implying that there was an average 10% increase in the probability of convulsions when dose increased by 1 mg/kg. The latter dose effect is equivalent to (1.1^25 ^= 10.8) an approximately 11-fold increase in the probability of convulsions when dose increased from 25 mg/kg to 50 mg/kg or from 50 mg/kg to 75 mg/kg. These results are consistent with the observed magnitude of the convulsive effects of the treatments (hydroxybupropion HCl > threohydrobupropion HCl > erythrohydrobupropion HCl > bupropion HCl) and the dose-dependent increase in the percentage of convulsing mice (primary outcome variable) with each treatment that was observed in this study.

**Table 2:** Hazard ratios from the Cox proportional hazards model comparisons of the time to onset of convulsions obtained for bupropion metabolites and bupropion HCl in mice

	**Predictors**	**Bupropion HCl**	**Erythrohydrobupropion HCl**	**Threohydrobupropion HCl**
Hydroxybupropion HCl*	Treatment	HR = 0.006 (p < 0.0001)	HR = 0.20 (p = 0.0002)	HR = 0.47 (p = 0.0310)
	Dose (25, 50, and 75 mg/kg)	HR = 1.10 (p < 0.0001)	HR = 1.09 (p < 0.0001)	HR = 1.11 (p < 0.0001)

*Hydroxybupropion HCl treatment was used as the control treatment for comparison because the number of events (convulsing mice/convulsions) in the bupropion HCl treatment was small and the hydroxybupropion HCl treatment induced convulsions in all 10 mice in the 50 and 75 mg/kg dose groups. HR, hazard ratio for the comparison of the treatment vs hydroxybupropion HCl.

### Convulsions per mouse

The mean ± SD convulsions per mouse and by dose group following the IP administration of treatment are shown in Table 3. Generally, there was a dose-dependent increase in the mean convulsions per mouse with the hydroxybupropion HCl treatment showing the highest values followed by threohydrobupropion HCl, erythrohydrobupropion HCl, and bupropion HCl with the lowest mean value. Results from an ANOVA model revealed that both treatment and dose were statistically significant (p < 0.0001 for each factor), indicating that the mean convulsions per mouse were statistically significantly different for the treatments as well as for the different doses.

**Table 3:** Mean ± SD convulsions per mouse following the intraperitoneal administration of bupropion HCl and bupropion metabolites

Dose* (mg/kg)	**Bupropion HCl**	**Erythrohydrobupropion HCl**	**Threohydrobupropion HCl**	**Hydroxybupropion HCl**
25	0	0	0	0
50	0	0.1 ± 0.3	7.0 ± 10.0	11.0 ± 10.3
75	0.5 ± 1.6	5.5 ± 6.2	21.2 ± 14.8	147.5 ± 106.0

*10 mice per dose per treatment.

### Duration of convulsions

Within each treatment and dose group, there was a consistent trend in the duration of convulsions observed. The mean number of short convulsions was the highest followed by the mean number of medium convulsions and then the mean number of long convulsions (Table 4). Similarly, with each treatment, there was a dose-related increase in the number of short, medium and long convulsions with the 75 mg/kg dose showing the largest mean number of convulsions of each subduration. Between the treatments, the hydroxybupropion HCl treatment showed the largest number of short, medium and long convulsions followed by threohydrobupropion HCl, erythrohydrobupropion HCl, and then bupropion HCl (Table 4).

**Table 4:** Mean ± SD number of short, medium, and long convulsions following the intraperitoneal administration of bupropion HCl and bupropion metabolites

Dose* (mg/kg)	**Bupropion HCl**			**Erythrohydrobupropion HCl**			**Threohydrobupropion HCl**			**Hydroxybupropion HCl**		
	
	**Short (0–10 s)**	**Medium (11–30 s)**	**Long (≥ 31 s)**	**Short (0–10 s)**	**Medium (11–30 s)**	**Long (≥ 31 s)**	**Short (0–10 s)**	**Medium (11–30 s)**	**Long (≥ 31 s)**	**Short (0–10 s)**	**Medium (11–30 s)**	**Long (≥ 31 s)**
**25**	-	-	-	-	-	-	-	-	-	-	-	-
**50**	-	-	-	0.1 ± 0.3	-	-	6.6 ± 8.9	0.3 ± 0.5	0.1 ± 0.3	7.9 ± 8.3	1.6 ± 2.6	1.5 ± 1.6
**75**	0.5 ± 1.6	-	-	4.4 ± 6.0	0.6 ± 1.0	0.5 ± 0.5	13.2 ± 9.7	5.5 ± 6.6	2.5 ± 2.3	136.1 ± 108.2	8.0 ± 7.2	3.4 ± 3.3

*10 mice per dose per treatment.

- Indicates no convulsions.

### Intensity of convulsions

Overall, the results obtained for the intensity of convulsions followed a consistent and similar trend to the results obtained for the duration of convulsions. Within each treatment and dose group, the mean number of mild convulsions was the highest followed by the mean number of moderate and then severe convulsions (Table 5). Similarly, with each treatment, there was a dose-related increase in the number of mild, moderate and severe convulsions with the 75 mg/kg dose showing the largest mean number of convulsions of each type of intensity. Between the treatments, the hydroxybupropion HCl treatment showed the largest number of mild, moderate and severe convulsions followed by threohydrobupropion HCl, erythrohydrobupropion HCl, and then bupropion HCl (Table 5).

**Table 5:** Mean ± SD number of mild, moderate and severe convulsions following the intraperitoneal administration of bupropion HCl and bupropion metabolites

Dose* (mg/kg)	**Bupropion HCl**			**Erythrohydrobupropion HCl**			**Threohydrobupropion HCl**			**Hydroxybupropion HCl**		
	
	**Mild**	**Moderate**	**Severe**	**Mild**	**Moderate**	**Severe**	**Mild**	**Moderate**	**Severe**	**Mild**	**Moderate**	**Severe**
**25**	-	-	-	-	-	-	-	-	-	-	-	-
**50**	-	-	-	-	0.1 ± 0.3	-	5.4 ± 8.9	1.3 ± 1.5	0.3 ± 0.7	7.0 ± 7.5	2.4 ± 2.3	1.6 ± 3.7
**75**	0.5 ± 1.6	-	-	3.4 ± 5.4	1.8 ± 2.0	0.3 ± 0.5	10.8 ± 7.5	5.7 ± 5.5	4.7 ± 6.1	113.3 ± 110.1	20.7 ± 20.2	13.3 ± 17.8

*10 mice per dose per treatment.

- Indicates no convulsions.

## Discussion

The administration of bupropion HCl is known to be associated with a dose-dependent risk of convulsions in both animals [17,18] and humans [2-9]. In addition, following the chronic administration of bupropion HCl to steady-state levels, it is known that bupropion metabolites are present in the plasma and cerebrospinal fluid in concentrations that are several times the concentration of the parent drug in both animals [19,20] and humans [2-4,12]. Nonetheless, the mechanism by which bupropion induces seizures remains unknown. More recently, there have been studies of the convulsive and anticonvulsive effects of bupropion HCl in mice [17,18]. However, there are no studies investigating the relative contribution of the individual metabolites and/or parent drug to the convulsive effects of bupropion HCl. Therefore, this experimental study was designed to investigate the convulsive liability of individual bupropion metabolites administered alone in mice.

The results of this study demonstrate that the IP administration of single doses of bupropion metabolites (erythrohydrobupropion HCl, threohydrobupropion HCl and hydroxybupropion HCl) are associated with a dose-dependent increase in the percentage of convulsing mice between 25 to 75 mg/kg. Hydroxybupropion HCl treatment producing the largest convulsive effect followed by threohydrobupropion HCl, erythrohydrobupropion HCl, and then bupropion HCl. Thus, all of the metabolites are more 'pro-convulsive' than the parent compound. Furthermore, the dose-response curves for the four treatments were statistically significantly different with CD_50 _values of 35, 50, 61 and 82 mg/kg for the hydroxybupropion HCl, threohydrobupropion HCl, erythrohydrobupropion HCl, and bupropion HCl treatments, respectively. It is noteworthy that the CD_50 _value obtained in this study for the bupropion HCl treatment is lower than the value of 120 mg/kg published earlier by Tutla *et al*. [17] for this experimental model. The reason for this discrepancy is probably due to the fact that the dosage range of 25 to 75 mg/kg used in this study does not include the previously reported CD_50 _for bupropion HCl [17] and only one mouse had convulsions in the bupropion HCl 75 mg/kg dose group. It is therefore, unlikely that the CD_50 _can be estimated accurately for bupropion HCl from this study.

Overall, the results of the secondary outcome variables were consistent with the results of the primary outcome variable in showing a dose-dependent increase in the observed convulsions, the probability of convulsions, and in the magnitude of the convulsive effects of the treatments with the hydroxybupropion HCl treatment producing the largest effect followed by threohydrobupropion HCl, erythrohydrobupropion HCl, and then bupropion HCl. In regard to the dose-dependent convulsive effects: (1) the analysis of the times to onset of first convulsion using the Cox proportional hazards model showed that there was an approximately 11-fold increase in the probability of convulsions when the dose increased from 25 to 50 mg/kg or from 50 to 75 mg/kg with each treatment; (2) the mean convulsions per mouse showed a dose-dependent increase across all treatments; and (3) both duration and intensity of convulsions were consistent in showing a dose-dependent increase in each subtype of convulsion with most convulsions short and mild and a fewer number of convulsions long and severe, respectively. Secondly, with respect to the magnitude of the observed convulsive effects, from the Cox proportional hazards model, the probability of convulsions were 99.4%, 80% and 53% significantly less likely with the bupropion HCl, erythrohydrobupropion HCl, and threohydrobupropion HCl treatments, respectively, compared to the hydroxybupropion HCl treatment. In addition, the mean convulsions per mouse, the number of short, medium and long convulsions, and the number of mild, moderate and severe convulsions were all consistently largest with the hydroxybupropion HCl treatment followed by threohydrobupropion HCl, erythrohydrobupropion HCl, and then bupropion HCl. In addition, both mortality and the observed clinical signs in mice followed the same dose-dependent and metabolite-dependent trends as the outcome variables. This consistency in the observed and analyzed results underscores the robustness of the results of this study.

Although equimolar concentrations of the metabolites to the concentrations of bupropion HCl 25, 50 and 75 mg/kg were used in the study, it is known that in humans following the chronic administration of bupropion HCl, these metabolites are present in the plasma in concentrations that are several times that of the parent drug [2-4]. The results of this study reveal that the convulsive liability of hydroxybupropion HCl is highest followed by threohydrobupropion HCl, erythrohydrobupropion HCl, and then bupropion HCl, and is consistent with the reported trend in the magnitude of their AUCs at steady-state plasma levels following chronic dosing with bupropion HCl in humans [2-4]. The latter further suggests that the metabolites may contribute significantly to the convulsive effects of bupropion HCl since bupropion-induced convulsions are known to be dose-dependent [2-9] and hence, concentration-dependent. However, a limitation of this study is that the convulsive liability of the parent drug was not evaluated and secondly, due to differences between animal and human metabolism of bupropion, results of *in vivo *animal studies may not translate to humans [14].

## Conclusion

The demonstration of the convulsive liability of the individual bupropion metabolites in this study is a novelty. Our results showed that bupropion metabolites dose-dependently increased the percentage of convulsing mice within the dosage range studied with hydroxybupropion HCl producing the largest effect followed by threohydrobupropion HCl, erythrohydrobupropion HCl, and then bupropion HCl. The dose-response curves were significantly different between the individual treatments and the CD_50 _values were, respectively, 35, 50, 61 and 82 mg/kg. The probability of inducing convulsions were 99.4%, 80% and 53% significantly less likely with bupropion HCl, erythrohydrobupropion HCl and threohydrobupropion HCl, respectively, compared to hydroxybupropion HCl in the dosage range studied. The probability of convulsions, the mean convulsions per mouse, the numbers of the three durations and three intensities of convulsions all increased with dose, and also, confirmed the metabolite-dependent trend of the effect (hydroxybupropion HCl > threohydrobupropion HCl > erythrohydrobupropion HCl > bupropion HCl) observed with the primary outcome variable. The finding that the convulsive liabilities of the metabolites are consistent with the magnitude of their AUCs following steady-state dosing in humans further suggests that they may contribute significantly to the convulsive effects of bupropion HCl.

## Competing interests

The authors declare that they have no competing interests.

## Authors' contributions

PHS participated in the design of the study and drafted the manuscript. RW participated in the design of the study and its coordination. LM participated in the design of the study and its coordination. RF performed the statistical analysis. SF participated in the design of the study and its coordination. All authors read and approved the final manuscript.
